# Adherence to the Mediterranean Diet and Environmental Impact of the Diet on Primary School Children Living in Parma (Italy)

**DOI:** 10.3390/ijerph17176105

**Published:** 2020-08-21

**Authors:** Alice Rosi, Beatrice Biasini, Michele Donati, Cristian Ricci, Francesca Scazzina

**Affiliations:** 1 Department of Food and Drugs, University of Parma, 43124 Parma, Italy; alice.rosi@unipr.it (A.R.); beatrice.biasini@unipr.it (B.B.); 2 Department of Chemistry, Life Sciences and Environmental Sustainability, University of Parma, 43124 Parma, Italy; michele.donati@unipr.it; 3 Pediatric Epidemiology, Department of Pediatrics, Medical Faculty, Leipzig University, 04103 Leipzig, Germany; Cristian.Ricci@medizin.uni-leipzig.de

**Keywords:** Mediterranean diet, children, sustainability, life cycle assessment, carbon footprint, ecological footprint, greenhouse gas emission, food diary, meat, fruit and vegetables

## Abstract

The key role of diet in both human health and environmental sustainability is well known. However, there is a lack of studies investigating the environmental impact of children’s dietary behavior. The aim of this observational study was to investigate the dietary environmental impact in a sample of primary school children living in Parma (Italy, *n* = 172, 8–10 years), in relation to their adherence to the Mediterranean diet (MD). Children completed a 3-day food record in both winter and spring. Dietary records were processed to obtain: (i) adherence to the MD and (ii) mean daily carbon and ecological footprints. Adherence to the MD was similar in winter and spring, with almost half of the participants showing a medium MD score. Carbon and ecological footprints were higher during winter, and the main dietary contributors were red and processed meat for both indexes. A small positive correlation was observed between adherence to the MD and total carbon and ecological footprints. This study provided the first analysis of the relationship between adherence to the MD and environmental impact of primary school children. Further research is needed to better investigate the environmental impact of primary school children’s diet and the possible relationship between the MD and environmental sustainability.

## 1. Introduction

The key role of diet on both human health and environmental sustainability has been widely investigated, with an increasing interest in ecological perspectives. Several indicators have been proposed to assess environmental impact of food and diets. Among these, the most used is represented by carbon dioxide-equivalent greenhouse gas (GHG) emissions, commonly referred to as carbon footprint, which accounts for CO_2_, N_2_O and CH_4_, followed by land, water and energy usage [1]. Anthropogenic GHG emissions are recognized as a leading driver of climate change, a critical planetary boundary that together with the increasing risk for biosphere integrity is threatening the Earth system [2]. According to the Intergovernmental Panel on Climate Change, the emissions of the global food system—defined as all the elements, activities and outputs involved from food production to consumption—are estimated to account for 21–37% of total net human-induced GHG emissions [3], contributing to detrimental effects on natural resources, such as land, soil, forestry heritage, water, biodiversity, as well as on nitrogen and phosphorous cycles [4]. Based on the One Health concept [5], human health and environmental health are strictly interconnected. They represent two of the pillars of diet sustainability, the conceptualization of which also encompasses cultural and social (food acceptability), as well as economic (food affordability and economic fairness) dimensions, as described by Food and Agriculture Organization of the United State (FAO) [6,7].

Globally, socio-demographic factors, such as urbanization and higher incomes, are associated with dietary shifts characterized by a higher intake of animal-based foods and a decreased consumption of plant-based protein sources, fruit and vegetables [8]. Indeed, considering eating patterns as a whole is more relevant than considering single food consumption, as no food is healthy per se [9]. Poor diets have been associated with an increased risk of cardio-metabolic diseases and cancer, negatively impacting on mortality and morbidity outcomes [10]. Nutrition is of paramount importance at every stage of life representing a leading risk factor in the global burden of disease. Furthermore, food habits established in childhood and youth result in long-term effects, setting the scene for dietary choices and eating behaviors in adulthood [11], thereby affecting health and well-being in later life [12].

Extensive literature has linked dietary patterns inspired by the Mediterranean diet (MD) to several health advantages, ranging from the prevention of cardiovascular diseases [13,14] and reduction in cancer risk [15] to an inverse association with depression and cognitive impairment [16] and a positive association with sleep quality [17]. The MD is generally associated with lower environmental impacts compared to alternative dietary patterns characterized by a higher consumption frequency of animal-based products [18]. From the nutritional and environmental perspectives, the MD is sustainable as it ensures adequate nutritional quality and respects biodiversity [19]. To date, a large number of studies have investigated the resource demand and GHG emissions of various healthy diets compared to current average diets [8,18,20]. Among these, the MD emerged as a sustainable dietary pattern, showing lower values in terms of CO_2_ equivalent emissions and land and water use [18]. Compared to forecast dietary impacts in 2050, projections estimate that shifting to plant-based diets could reduce global diet-related GHG emissions up to 60%, with a reduction in the future crop-land use of up to 35% within a production system requiring lower inputs of fertilisers [20]. The MD is a plant-based diet showing strong similarities with the “healthy diets from sustainable food systems” defined by the EAT Lancet Commission [4]. Both dietary approaches are characterized by daily intakes of (i) complex carbohydrates derived from whole grains, fruit and vegetables, and nuts; (ii) a preference for sources of unsaturated fatty acids; and (iii) limited consumption of red and processed meat as well as refined grains. Based on these considerations, it is possible to consider the MD as an example of a healthy and sustainable diet in those contexts where adequate levels of food security, affordability, accessibility and cultural acceptability are satisfied.

Although the analyses of the environmental impact of food and diet have been attracting increased attention in the last years, to the best of our knowledge, there is no evidence of environmental performance of children’s dietary patterns. Moreover, information on the relationship between adherence to the MD and environmental impacts of the actual diet of young populations is not available in the literature. Therefore, the present empirical study addresses this literature gap investigating the environmental impact of children’s diet in relation to their adherence to the MD in a sample of primary school children living in Parma (Italy). In particular, the aims of the study were: (i) to assess the adherence to the MD diet; (ii) to evaluate the environmental impact in terms of GHG emissions and land use; and (iii) to investigate the relationship between adherence to the MD and the carbon and ecological footprints of actual diet investigated during both winter and spring.

## 2. Materials and Methods

### 2.1. Subjects and Study Design

Subjects and study design have been described elsewhere [21]. Briefly, 220 third and fourth grade primary school children (8–10 years old) enrolled in the Giocampus school program, an educational project aiming to promote physical activity and healthy eating among primary school children in Parma) [22], were asked to participate in this empirical study.

Parents/legal guardians and teachers were informed about the study protocol and were guaranteed complete anonymity. Parents/legal guardian were asked to provide their written informed consent. Children who had the informed consent signed by parents/legal guardians were informed about the study and were asked to give their oral informed assent, which was registered in a specific data collection sheet. The study was performed in compliance with the Declaration of Helsinki and was approved by the local Ethical Committee (AZOSPR Prot. n. 5351_15022016).

Dietary data were collected during three consecutive days in winter (December–January) and three consecutive days in spring (April–May) in the same school year using a 3-day dietary record adapted for children. Each child, with the help of parents and teachers, recorded all food and beverages consumed during the assessment days, with their relative portion sizes. The portion size was weighed whenever possible, as in the case of meals consumed at home, or estimated, as in the case of meals consumed at school, knowing the standard portion size served to children and considering the possible leftover on the plate.

### 2.2. Adherence to the Mediterranean Diet

Mean daily intakes of food groups were estimated from the food diaries and were used to evaluate the adherence to the MD through a revised version of the Mediterranean diet score (MDS) developed for adults by Trichopoulou and colleagues [23] and adapted for the paediatric population enrolled in the Healthy Lifestyle in Europe by Nutrition in Adolescence (HELENA) study [24]. The original version of the MDS is composed of seven beneficial components (vegetables, legumes, fruits and nuts, cereals, fish, monounsaturated fat:saturated fat ratio, and wine) and two detrimental components (meat and poultry, and dairy products). In the modified version, considering the recommendation for the paediatric population, dairy products were considered as a positive component while alcohol as a negative component. A value of 0 or 1 point was assigned to each component using as cut-off values the median intakes of the studied population. For the positive food groups, 1 point if the intake was higher than the median value and 0 point if the intake was lower. For detrimental components, 1 point was assigned if the intake was lower and 0 point if the intake was higher than the median value. The total MDS ranged from 0 to 9 points, and it was classified into 3 levels of adherence to the MD: low (score 0–3 points), medium (4–5 points), and high (6–9 points).

### 2.3. Carbon and Ecological Footprint Evaluation

The dietary environmental impact was calculated taking into account two of the most representative indexes of the agri-food system [25]: the carbon footprint (g of CO_2_ equivalent emissions) and the ecological footprint (m^2^ of land needed to regenerate the applied resources). Food data from the 3-day diaries were matched to those included in the environmental impact database of the Barilla Centre for Food and Nutrition [26], from which the two environmental indexes were retrieved. The mean daily carbon footprint (g CO_2_ eq/day) and ecological footprint (m^2^/day) were estimated for each child, taking into account the intake of each food item during the three assessment days recorded in the food diaries. Recipes and multi-ingredients foods were broken down into raw foods considering their corresponding proportions. For cooked food, environmental values took into consideration the cooking methods (e.g., boiling, deep frying, baking). Environmental impacts were calculated for the total diet, for food sources (e.g., animal-based food, vegetable-based food) and for specific food groups (e.g., meat, milk and dairies, fruit, legumes, vegetables) by summing the impacts of single food items.

### 2.4. Statistical Analysis

IBM SPSS statistics for Macintosh Version 26.0 (IBM Corp., Armonk, NY, USA) was used to perform all the statistical analyses, keeping the significance at *p* < 0.05. The normality of data distribution was assessed through the Kolmogorov–Smirnov test. Differences between winter and spring were explored using a paired sample *t*-test for normally distributed variables (energy and food group intakes, total and main food sources environmental impacts) or a non-parametric Wilcoxon signed-ranks test for paired samples for not normally distributed variables (MDS scores and environmental impacts from single food groups). Differences among adherence to the MD groups within each season were explored thorough a one-way analysis of variance (ANOVA) with a Bonferroni post hoc test for normally distributed variables or a Kruskal–Wallis non-parametric one-way ANOVA for independent samples with multiple pairwise comparisons for not normally distributed variables. Last, the relationship between adherence to the MD and carbon and ecological footprints was investigated using the Spearman’s rank correlation coefficient irrespective of the season. Since the environmental impacts of diet increase with the caloric intake [27], the same between-season and within-MD group analyses were performed using energy-adjusted data to assess differences in environmental impacts per 1000 kcal.

### 2.5. Power Calculation and Sample Size Justification

Numerous power calculations were performed to evaluate sample representativeness and type-II error rates in relation to the sample size. Firstly, for sample representativeness, we considered that the total population of primary school students in Parma aged 8–10 years is composed of 2500–3000 subjects. We then estimated that the study sample should be composed of at least 110–130 children (90% level of confidence and 10% marginal error) to be representative of the study population. Afterwards, we considered standardized effect sizes in the form of Cohen’s d for paired and unpaired *t*-tests and Cohen’s ƒ^2^ for ANOVA to evaluate the statistical power of comparisons aimed to evaluate the environmental impact in terms of GHG emissions and land use. According to these calculations, we report that a sample size of 40–80 individuals per group was sufficient to detect a large to medium effect size for a two independent samples *t*-test with a statistical power of 80% (d = 0.63–0.45 for n = 40–80, α = 0.05, and d = 0.74–0.52 for n = 40–80, α = 0.017 considering Bonferroni adjusted type-I error rate). Moreover, a sample size of 40–80 subjects was sufficient to detect a medium effect size on the dependent samples *t*-test (d = 0.45–0.32 for n= 40–80, α = 0.05 b). The same sample size adopted here resulted in medium effect size when considering the ANOVA F effect size for the omnibus test with a statistical power of 80% (F = 0.29–0.20 for n = 40–80, α = 0.05). Finally, to investigate the relationship between adherence to the MD and the carbon and ecological footprints of actual diet investigated during both winter and spring, we performed a power calculation based on Spearman’s correlation coefficients. Here, a sample size of 40 subjects is sufficient to detect a correlation coefficient above 0.43 (α = 0.05, 1 − β ≥ 0.8). The above evaluations were confirmed when considering the analogous non-parametric tests. Finally, a post hoc power calculation was performed, and type-II error rate was added for borderline non-significant results (0.1 > *p* > 0.05).

## 3. Results

### 3.1. Participants’ Adherence to the MD

Completed data were obtained from 172 children (52% females, 48% males, 8–10 years old), and a total of 344 3-day food diaries were analysed. Participants’ diets have been already fully described in a previous paper [21], and lower intakes for food groups, energy and nutrients were reported in spring rather than winter. When food intakes were computed for the major food sources (Table 1), a decrease in food consumption from winter to spring was registered for plant-based food (e.g., vegetables, legumes, potatoes, cereals, bread, fruit, nuts, juices, *p* < 0.001), mixed food (e.g., beverages, pizza, sweets and desserts, *p* < 0.001), and beverages (e.g., soft drink, *p* = 0.026), while between-season differences were not observed for the quantity of animal-based food consumed (e.g., meat, poultry, cured meat, fish, eggs, milk, dairies).

The MDSs were similar between seasons corresponding to medium values of adherence to the MD (Table 1). Out of the total children, 27% and 23% showed a low adherence, 46% and 44% a medium adherence, and 27% and 33% a high adherence to the MD, respectively, during winter and spring. Children in the high adherence to the MD group had a higher intake than participants in the low adherence group of all beneficial components considered for the evaluation of the MDS in both seasons (Table S1), except for cereals, which were comparable between groups in winter. The intakes of the detrimental food groups (e.g., meat) were similar among adherence to the MD groups in both seasons.

### 3.2. Carbon and Ecological Footprint

The carbon and ecological footprints are presented in Table 2 for winter and spring.

The total carbon footprint was slightly higher in winter than in spring (*p* = 0.044), and the total ecological footprint decreased from winter to spring (*p* = 0.001). In contrast, when environmental data were expressed per 1000 kcal, no significant differences were observed between seasons for both total carbon and ecological footprints.

Considering the food sources, impacts were higher in winter than in spring for both environmental indicators in the case of plant-based food (*p* = 0.006 and *p* = 0.004, respectively, for the carbon and the ecological footprints), mixed food (*p* = 0.001 for both footprints), and beverages (*p* = 0.035 and *p* = 0.002, respectively, for the carbon and ecological footprints). Considering the environmental indicators per 1000 kcal, values were similar between seasons except for the carbon footprint from animal-based food that increased in spring (*p* = 0.001).

**Figure 1:**
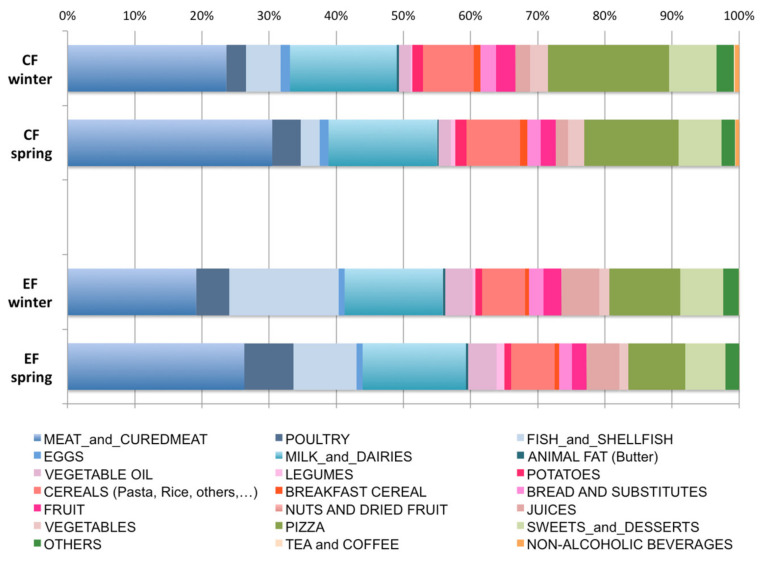
Relative contribution (%) of food groups to total carbon footprint (CF) and ecological footprint (EF) by seasons. Blue gradients: animal-based food; pink gradients: plant-based food; green gradients: mixed food; orange gradients: beverages.

The contribution to total carbon and ecological footprints of food sources and of major food groups is showed in Figure 1.

Animal-based products represented around 50% of the impact on the carbon footprint during winter and more than half (55–60%) of the carbon footprint during spring and the ecological footprint in both seasons. In contrast, plant-based food had lower impacts (22–24% for both indicators). Similarly, mixed food impacts ranged from 16% (spring ecological footprint) to 28% (winter carbon footprint). Beverages had a very low relative impact (0–1%) for both indicators and in both seasons. When considering single food groups or dishes, meat and cured meat products were the major contributors for both environmental indicators and in both spring and winter, ranging between 19–31% of the total impacts, followed by milk and dairies (15–16%). The third major contributor to the carbon footprint was pizza (14–18%), while fish and shellfish represented 9–16% of the ecological footprint.

A more detailed analysis of the environmental impacts of specific food groups or dishes is presented in Tables S2 and S3.

### 3.3. Relationship between the MD and Environmental Impacts

A one-way between-groups analysis of variance was conducted to explore differences among the groups of adherence to the MD in terms of environmental impacts (Table 3).

A statistically significant difference was found in both seasons in the total carbon footprint (*p* = 0.020 and *p* = 0.005, respectively, for winter and spring) and in the total ecological footprint (*p* < 0.001 for both seasons). Post hoc comparisons among groups indicated that the carbon footprint was higher for the high adherence to the MD group than the low adherence group, while the mean ecological footprint was higher for the high adherence MD groups than the low and medium adherence groups. Similarly, significant differences were observed for the carbon footprint derived from the plant-based food (*p* < 0.001 for both seasons) and for the ecological footprint derived from both animal- (*p* < 0.001 for winter and *p* = 0.005 for spring) and plant-based food (*p* < 0.001 for both seasons), with participants in the high adherence to the MD group showing higher impacts for the two environmental indicators.

In contrast, when environmental data were expressed per 1000 kcal, no significant differences in the carbon footprint were observed among adherence to the MD groups in both seasons, except for the impact derived from the plant-based food (*p* < 0.001 for winter, and *p* = 0.027 for spring), which was lower in the low adherence group than the other groups in winter and only compared to the high adherence group in spring. Statistically significant differences were found in the total (*p* = 0.009), animal-based (*p* = 0.033) and plant-based (*p* = 0.011) ecological footprint per 1000 kcal, with higher values found in the high adherence MD group in winter, while the ecological footprints were similar among the adherence groups in spring. A detailed analysis of differences among adherence to the MD groups and carbon and ecological footprints due to single food groups is presented in Tables S4 and S5.

The relationship between adherence to the MD and environmental impacts was also explored using correlation coefficients irrespective of the season (Table 4).

A small significant correlation was observed between the MDS and total (*p* < 0.001) and animal-based (*p* = 0.008) carbon footprints, while a medium positive correlation was found for the carbon footprint from plant-based food (*p* < 0.001). In contrast, there were no correlations between MDS and carbon footprints when the environmental indicator values were obtained per 1000 kcal, except for a small–medium correlation between carbon footprint/1000 kcal and plant-based food (*p* < 0.001). For the ecological footprints, small–medium correlations were found for total (*p* < 0.001), animal-based (*p* < 0.001), and plant-based (*p* < 0.001) impacts. Small correlations were also discovered for values per 1000 kcal from total (*p* < 0.001), animal-based (*p* = 0.001), and plant-based food (*p* < 0.001).

A strong positive correlation was found between the two environmental indicators (data not shown) considering both values for the total diet (ρ = 0.855, *p* < 0.001) and values for 1000 kcal (ρ = 0.749, *p* < 0.001). Strong positive correlations were also found between carbon and ecological footprints for animal-based food (ρ = 0.893, *p* < 0.001), plant-based food (ρ = 0.859, *p* < 0.001) and other mixed food (ρ = 0.983, *p* < 0.001) and for values per 1000 kcal from animal-based food (ρ = 0.885, *p* < 0.001), plant-based food (ρ = 0.815, *p* < 0.001) and other mixed food (ρ = 0.981, *p* < 0.001). Impacts from beverages showed small–medium correlations for both pure values (ρ = 0.305, *p* < 0.001) and values per 1000 kcal (ρ = 0.296, *p* < 0.001).

## 4. Discussion

To the best of our knowledge, this is the first study that provides a comprehensive overview of the dietary environmental impact in terms of carbon and ecological footprints of primary school children living in Parma considering between-season differences and the association between environmental indicators and adherence to the MD. The results of the present study highlight some discrepancies in children’s food intake comparing the two seasonal data-collecting periods characterized by different eating patterns, with lower quantities of plant-based and mixed food consumed in spring. In spring, the proportion of children with a high adherence to the MD increased, while a lower percentage of children was found in the low adherence group. However, no differences in the average MDS were observed between seasons, with children showing an MDS corresponding to medium adherence to the MD in both periods. These results are consistent with previous reports that young populations in Mediterranean countries have modified their traditional food habits, orienting consumption towards globalized diets more rich in refined grains, saturated fat and sugars, with social, cultural and economic factors playing a role in this transition [28]. By comparing the present results with those found previously in similar children populations living in Parma, similar MDS can be observed both in subjects from 9 to 11 years of age (6.5 ± 2.2) [29] and from 11 and 13 years old (6.0 ± 2.3) [30]. The obtained medium levels of adherence were, however, assessed differently by applying the Mediterranean diet quality index for children and adolescents (KIDMED), which provides a score comprised in a range of from 0 to 12 points [31] instead of the 0–9 points system applied in the present study. Furthermore, by administering the KIDMED questionnaire, comparable medium levels (5.5 ± 2.1) were found in a children sample of a wider age range (6–16 years) living in Novara in northern Italy [32].

A thorough comparison with other data previously reported is not straight forward because, as far as we know, no studies on the environmental impact of school-age children have been reported in the literature and several differences can be found when comparing children’s diet with those of adults. The environmental assessment carried out by Germani and colleagues [25] on the food consumption of the Italian adult population demonstrated that a shift in the current dietary intake towards the Mediterranean pattern would result in a lower weekly environmental impact in terms of carbon, water and ecological footprints. Compared to the present results, in a study performed on the actual food consumption of young adults (18–20 years) also living in Parma, higher GHG emissions (approximately 2.4 kg of CO_2_ eq/person/day) and ecological footprint (EF) data (approximately 14.8 m^2^/person/day) were computed, although the food intake was lower (approximately 790 g of food per day) [33]. This is due to a different share of animal-based products, in particular meat, which accounts for about 45% of total food intake in young adults, while it did not exceed 35% of total food intake in the present study with children. It is noteworthy that a relevant contribution to food consumption, and consequently to the associated environmental impacts, is due to pizza, which is highly represented in the diets of both children and young adult samples. Similar to what was observed for primary school children, animal-based foods, and in particular meat and fish, were the main contributors to the dietary environmental footprints of an Italian adult population of omnivores [34]. In this adult population, both the total food intake (approximately 1.5 kg/day) and the carbon and ecological footprints were higher (approximately 4.0 kg of CO_2_ eq/person/day and 26.0 m^2^/person/day, respectively), while a medium adherence to the MD was reported [34]. In contrast, environmental impacts similar to those observed in the present study were observed in an Italian adult population consuming a vegan diet (2.3 of CO_2_ eq/person/day and 14.5 m^2^/person/day, respectively for the carbon and ecological footprints) and a high adherence to the MD [34].

Recent studies depicted a positive association between adherence to the MD and environmental performance of diet in adult Italian [35] and Spanish [36] populations. In contrast to what was expected, children consuming a diet characterized by a high MDS showed higher CO_2_ eq emissions when considering total food consumption and plant-based food intake, in winter and spring. Similarly, a higher ecological footprint was observed in children in the highest MDS group than in children in the lowest MDS group, for the total food consumption, plant-based food and animal-based food intakes in both seasons. It is worth mentioning that when the same assessments were made for energy-adjusted values, almost all such differences were not apparent. Therefore, the higher quantity of food consumed by the subjects who were more adherent to the MD can explain why their diet was more environmentally demanding. This finding is in line with previous evidence that dietary GHG emissions increase proportionally with energy intake [27]. Nevertheless, to ensure good health, eating less for environmental reasons is not a solution if the food consumption does not meet the recommended energy and nutrient values [27]. A trade-off between maintaining health and the environmental dimension of human diets has been reported in the literature. In this regard, a newly published systematic review found that adhering to the national dietary guidelines does not ensure eco-friendlier diets compared to the current average US diet [37]. Similarly, an improvement in the environmental performance was not necessarily associated with diet adequacy in terms of energy and nutrients considering national data of the adult populations of five European countries (Finland, France, Italy, Sweden, and the UK) [38]. With the aim of limiting the dietary environmental impact and attaining a high level of adherence to the MD, preference should be given to fresh and seasonal fruit and vegetables, as well as protein-based foods of vegetal origin, taking into account agricultural and processing techniques [34,38].

The present study provides an analysis of adherence to the MD, as well as an assessment of the environmental impacts in terms of GHG emissions and land requirement and of the relationship between the adherence to the MD and the environmental performance of actual diets in a sample of schoolchildren living in Parma. As a consequence, this approach is able to consider the peculiarities of a real-life dietary scenario and individual food preferences, unlike studies based on hypothetical dietary patterns [9]. Indeed, the use of two 3-day diaries completed in winter and in spring ensures the collection of complete and real food consumption data. In addition, the study applies multiple environmental impact indicators for a better evaluation of the environmental sustainability dimension associated with diet. However, extending the analysis to other environmental outcomes linked to food production would entail a wider assessment. For example, evaluating the dietary impact on biodiversity loss (extinction rate) and freshwater use, as well as fertilizer use (nitrogen and phosphorous application), would provide meaningful information about the environmental performance of diets within planetary boundaries [4,39]. When interpreting the results presented in this study, some limitations should be considered. Environmental information was obtained from a database that has some intrinsic limitations due to incomplete data, so a more detailed analysis was not possible. The study missed important considerations about the seasonality of food and the local or not local production. In this light, the between-season differences observed were due to the different food consumed by children, allowing only speculation on the seasonality of food consumed. Moreover, the environmental analysis was performed with food consumption and did not consider the environmental impact of leftovers and not consumed food. Differences in the environmental dataset applied could make it difficult to compare results among studies using different methodologies. Another point worth mentioning is the instrument used to evaluate the adherence to the MD. This information was obtained from food diaries using the MDS [23] adapted for the paediatric population [24], a specific and well-established tool for this purpose that uses the quantity of consumed food for adherence calculation. Other tools commonly used, such as the KIDMED questionnaire [31], estimate the adherence through frequency of consumption. Therefore, in this study, the adherence to the MD was strongly dependent on the consumption of the beneficial food groups considered in the MDS (e.g., vegetables, legumes, fruits and nuts, cereals, fish), leading to an increase in the environmental impacts linked to the higher intake of these food groups. Evaluating the adherence to the MD with a different tool may have led to slightly different results. The other limitation of the present study is the relatively small sample size recruited in a narrow geographic area that could not be considered representative of the Italian schoolchildren. Moreover, we cannot exclude that our limited sample size might have determined false negative results due to the lack of statistical power. According to our post hoc power analyses conducted on borderline statistical results, we can consider this limitation as negligible.

## 5. Conclusions

In conclusion, a deep gap in the scientific literature on the environmental impact of youth has been observed. This work helps to better understand what main food groups are contributing to the environmental impact of the diet of Italian primary school children in different seasons. Carbon and ecological footprints were found to be higher during winter, the main dietary contributor being red and processed meat for both environmental indicators. The relationship between the adherence to the MD and the carbon and water footprints was also assessed. Irrespectively of seasonality, a weak correlation was observed between the adherence to the MD and total carbon and ecological footprints. Further research is needed to shed light on the environmental impact of primary school children’s diet throughout the year in other cohorts. Particular emphasis might be placed on the importance of the overall food intake on the environmental impact, rather than the consumption of specific food groups.

## Figures and Tables

**Table 1:** Daily intakes of main food sources and adherence to the Mediterranean diet (MD) by seasons.

Source	Winter g/day	Spring g/day	*p* ^1,2^
Total intake	995 ± 239	864 ± 235	<0.001 ^1^
Animal-based food	240 ± 82	229 ± 89	0.088 ^1^
Plant-based food	487 ± 170	421 ± 173	<0.001 ^1^
Mixed food	201 ± 110	163 ± 83	<0.001 ^1^
Beverages	64 ± 86	48 ± 83	0.026 ^1^
Adherence to the MD (MDS) ^3^	4.5 (3.0–6.0)	5.0 (4.0–6.0)	0.174 ^2^

^1^ Between-season differences (paired sample *t*-test, *p* < 0.05; normally distributed variables). Values are mean ± SD. Animal-based food (i.e., meat, poultry, cured meat, fish, eggs, milk, dairies); plant-based food (i.e., vegetables, legumes, potatoes, cereals, bread, fruit, nuts, juices); mixed food (i.e., pizza, filled pasta, sweets and desserts); beverages (i.e., soft drink; tea and coffee decaffeinated). ^2^ Between-season differences (non-parametric Wilcoxon signed-ranks test for paired samples, *p* < 0.05; not normally distributed variable). Values are median (25th–75th percentile). ^3^ MDS: Mediterranean diet score. MDS ranged from 0 to 9 points. Values ≤3 points indicate low adherence to the MD, values between 4 and 5 points indicate medium adherence and values ≥6 points indicate high adherence.

**Table 2:** Total and food-source carbon footprint (CF) and ecological footprint (EF) by seasons per day and by seasons per day per 1000 kcal of total diet.

**Source**	**CF Winter** **g CO_2_ eq/day**	**CF Spring** **g CO_2_ eq/day**	***p*** **^1^**	**EF Winter** **m^2^/day**	**EF Spring** **m^2^/day**	***p*** **^1^**
Total diet	2331 ± 650	2207 ± 695	0.044	14.9 ± 3.8	13.8 ± 4.4	0.001
Animal-based food	1151 ± 516	1222 ± 632	0.169	8.4 ± 3.4	8.2 ± 4.0	0.544
Plant-based food	520 ± 146	481 ± 165	0.006	3.7 ± 1.2	3.3 ± 1.4	0.004
Mixed food	646 ± 454	497 ± 402	0.001	2.9 ± 1.8	2.3 ± 1.6	0.001
Beverages	18 ± 26	13 ± 28	0.035	0.0 ± 0.01	0.00 ± 0.00	0.002
	**CF Winter** **g CO_2_ eq/1000 kcal**	**CF Spring** **g CO_2_ eq/1000 kcal**	***p*** **^1^**	**EF Winter** **m^2^/1000 kcal**	**EF Spring** **m^2^/1000 kcal**	***p*** **^1^**
Total diet	1480 ± 305	1545 ± 413	0.074	9.6 ± 2.0	9.7 ± 2.6	0.691
Animal-based food	738 ± 323	859 ± 431	0.001	5.4 ± 2.2	5.8 ± 2.7	0.141
Plant-based food	334 ± 85	338 ± 102	0.718	2.3 ± 0.7	2.3 ± 0.9	0.652
Mixed food	399 ± 270	344 ± 267	0.054	1.8 ± 1.0	1.6 ± 1.0	0.052
Beverages	10.9 ± 16.6	8.9 ± 19.3	0.078	0.0 ± 0.0	0.0 ± 0.0	0.002

^1^ Between-season differences (paired sample *t*-test, *p* < 0.05; all sources were normally distributed variables, except beverages, for which a non-parametric Wilcoxon signed-ranks test for paired samples was performed). Values are mean ± SD. Animal-based food (i.e., meat, poultry, cured meat, fish, eggs, milk, dairies); plant-based food (i.e., vegetables, legumes, potatoes, cereals, bread, fruit, nuts, juices); mixed food (i.e., pizza, filled pasta, sweets and desserts); beverages (i.e., soft drinks; tea and coffee decaffeinated).

**Table 3:** Total and food source carbon footprint (CF) and ecological footprint (EF) among adherence to the Mediterranean diet groups by seasons per day and by seasons per 1000 kcal.

Source	Low MDS (*n* = 46)	Medium MDS (*n* = 80)	High MDS (*n* = 46)	*p* ^1^	Low MDS (*n* = 39)	Medium MDS (*n* = 76)	High MDS (*n* = 57)	*p* ^1^
	**CF Winter g CO_2_eq/day**				**CF Spring g CO_2_eq/day**			
Total diet	2153 ± 723 ^a^	2319 ± 602 ^ab^	2528 ± 610^b^	0.020	1968 ± 687 ^a^	2164 ± 695 ^ab^	2427 ± 646 ^b^	0.005
Animal-based food	1070 ± 565	1125 ± 507	1278 ± 466	0.128	1168 ± 667	1185 ± 695	1307 ± 510	0.457
Plant-based food	419 ± 110 ^a^	526 ± 139 ^b^	690 ± 126^c^	<0.001	385 ± 120 ^a^	461 ± 150 ^b^	573 ± 167 ^c^	<0.001
Mixed food	650 ± 468	652 ± 473	634 ± 415	0.977	396. ± 314	512 ± 419	544 ± 427	0.187
Beverages	14 ± 24	20 ± 29	17 ± 23	0.590	19 ± 41	101 ± 23	12 ± 22	0.568
	**EF Winter m^2^/day**				**EF Spring m^2^/day**			
Total diet	13.2 ± 4.1 ^a^	14.7 ± 3.3 ^b^	17.2 ± 3.3^c^	<0.001	11.8 ± 4.1 ^a^	13.3 ± 4.5 ^a^	15.9 ± 3.6 ^b^	<0.001
Animal-based food	7.2 ± 37 ^a^	8.1 ± 3.2 ^a^	10.2 ± 2.9^b^	<0.001	7.3 ± 4.0 ^a^	7.7 ± 4.4 ^a^	9.6 ± 2.9 ^b^	0.005
Plant-based food	3.0 ± 1.0 ^a^	3.7 ± 1.2 ^b^	4.2 ± 1.1^c^	<0.001	2.6 ± 1.0 ^a^	3.2 ± 1.3 ^a^	3.9 ± 1.6 ^b^	<0.001
Mixed food	3.0 ± 1.9	2.9 ± 1.9	2.8 ± 1.6	0.931	1.9 ± 1.2	2.3 ± 1.7	2.6 ± 1.7	0.223
Beverages	0.00 ± 0.01	0.00 ± 0.01	0.00 ± 0.01	0.685	0.00 ± 0.00 ^ab^	0.00 ± 0.00 ^a^	0.00 ± 0.01 ^b^	0.016
**Energy-adjusted**	**CF Winter g CO_2_ eq/1000 kcal**				**CF Spring g CO_2_ eq/1000 kcal**			
Total diet	1444 ± 339	1501 ± 294	148 ± 290	0.602	1592 ± 501	1531 ± 425	1532 ± 326	0.728
Animal-based food	722 ± 353	77 ± 325	756 ± 295	0.878	940 ± 517	835 ± 460	834 ± 32	0.406
Plant-based food	289 ± 74 ^a^	345 ± 91 ^b^	360 ± 65^b^	<0.001	312 ± 93^a^	330 ± 107 ^ab^	365 ± 97 ^b^	0.027
Mixed food	422 ± 279	409 ± 292	360 ± 218	0.494	325 ± 255	361 ± 289	334 ± 248	0.746
Beverages	9.6 ± 15.8	12.1 ± 179	10.5 ± 15.2	0.743	147 ± 29	7.2 ± 15.7	7.3 ± 13.1	0.421
**Energy-adjusted**	**EF Winter m^2^/1000 kcal**				**EF Spring m^2^/1000 kcal**			
Total diet	9.0 ± 2.2 ^a^	9.6 ± 1.9 ^ab^	10.2 ± 1.7 ^b^	0.009	9.5 ± 3.1	9.4 ± 2.8	10.1 ± 2.0	0.297
Animal-based food	4.9 ± 2.4 ^a^	5.4 ± 2.2 ^ab^	6.1 ± 1.9 ^b^	0.033	5.8 ± 3.1	5.5 ± 2.9	6.1 ± 1.9	0.351
Plant-based food	2.0 ± 0.7 ^a^	2.42± 0.8 ^ab^	2.5 ± 0.5 ^b^	0.011	2.2 ± 0.9	2.3 ± 0.9	2.5 ± 0.9	0.243
Mixed food	1.9 ± 1.1	1.8 ± 1.1	1.1 ± 0.8	0.321	1.6 ± 1.0	1.6 ± 1.1	1.5 ± 1.0	0.761
Beverages	0.00 ± 0.01	0.00 ± 0.01	0.00 ± 0.00	0.668	0.00 ± 0.00 ^ab^	0.00 ± 0.00 ^a^	0.00 ± 0.01 ^b^	0.016

^1^ Differences among adherence to the MD groups within each season (one-way ANOVA with Bonferroni post hoc test, *p* < 0.05; all sources were normally distributed variables, except beverages, for which a Kruskal–Wallis non-parametric one-way ANOVA for independent samples with multiple pairwise comparisons was performed). Different letters in the same raw indicate significant differences among MD groups (a < b < c). Values are mean ± SD. Animal-based food (i.e., meat, poultry, cured meat, fish, eggs, milk, dairies); plant-based food (i.e., vegetables, legumes, potatoes, cereals, bread, fruit, nuts, juices); mixed food (i.e., pizza, sweets and desserts); beverages (i.e., soft drinks). MDS: Mediterranean diet score (low: 0–3 points; medium: 4–5 points; high: 6–9 points).

**Table 4:** Spearman’s correlation coefficients between MDS and carbon and ecological footprints due to total, animal-based, plant-based and other mixed food per day and per 1000 kcal.

		Total	Animal-Based Food	Plant-Based Food	Mixed Food	Beverages
		**Carbon footprint g CO_2_ eq/day**				
**MDS**	**ρ Spearman**	0.225	0.144	0.491	0.014	0.013
	***p*** **value**	<0.001	0.008	<0.001	0.789	0.805
		**Ecological footprint m^2^/day**				
**MDS**	**ρ Spearman**	0.363	0.298	0.354	0.004	0.056
	***p*** **value**	<0.001	<0.001	<0.001	0.935	0.302
		**Carbon footprint g CO_2_ eq/1000 kcal**				
**MDS**	**ρ Spearman**	−0.001	0.016	0.303	−0.074	−0.008
	***p*** **value**	0.990	0.761	<0.001	0.168	0.878
		**Ecological footprint m^2^/1000 kcal**				
**MDS**	**ρ Spearman**	0.190	0.172	0.199	−0.106	0.055
	***p*** **value**	<0.001	0.001	<0.001	0.059	0.311

## Supplementary Materials

The following are available online at https://www.mdpi.com/1660-4601/17/17/6105/s1, Table S1: Daily intake of the food groups considered for the MDS among adherence to the Mediterranean diet groups by seasons; Table S2: Carbon footprint (CF) and ecological footprint (EF) by seasons for each food groups; Table S3: Energy-adjusted carbon footprint (CF) and ecological footprint (EF) by seasons for each food groups; Table S4: Total and food-source carbon footprint (CF) and ecological footprint (EF) for each food groups among adherence to the Mediterranean diet groups by seasons; Table S5: Energy-adjusted (per 1000 kcal) total and food-source carbon footprint (CF) and ecological footprint (EF) for each food groups among adherence to the Mediterranean diet groups by seasons.
