# Supplementary material for: Adherence to the Mediterranean Diet and Environmental Impact of the Diet on Primary School Children Living in Parma (Italy)
Source: Int J Environ Res Public Health. 2020 Aug 21;17(17):6105. doi: 10.3390/ijerph17176105 (PMC7503352; doi:10.3390/ijerph17176105)
Supplement: Supplementary file 1 [file ijerph-17-06105-s001.pdf]

Supplementary Materials

Table S1. Daily intake of the food groups considered for the MDS among adherence to the Mediterranean Diet groups by seasons.

Source	Low MDS (*n* = 46)	Medium MDS (*n* = 80)	High MDS (*n* = 46)	*p* [1]	Low MDS (*n* = 39)	Medium MDS (*n* = 76)	High MDS (*n* = 57)	*p* [1]
	**Winter**				**Spring**			
Vegetable (g/day)	50 (22–83) [a]	88 (50–133) [b]	140 (109–169) [c]	<0.001	56 (29–73) [a]	68 (40–115) [b]	112 (91–140) [c]	<0.001
Legumes (g/day)	0.0 (0.0–0.0) [a]	0.0 (0.0–0.0) [ab]	0.0 (0.0–10.8) [b]	0.021	0.0 (0.0–0.0) [a]	0.0 (0.0–10.0) [ab]	1.7 (0.0–16.7) [b]	0.006
Fruits and nuts (g/day)	65 (30–105) [a]	105 (63–183) [b]	183 (148–233) [c]	<0.001	48.2 (1.9–103.0) [a]	68.2 (21.3–116.8) [a]	131.9 (88.7–184.7) [b]	<0.001
Cereals (g/day)	225 (163–274)	241 (181–287)	253 (177–299)	0.239	163 (151–186) [a]	206 (171–255) [b]	228 (175–275) [b]	<0.001
Fish and seafood (g/day)	6.7 (0.0–30.0) [a]	30.0 (0.0–39.0) [a]	48.8 (30.0–70.4) [b]	<0.001	0.0 (0.0–0.0) [a]	0.0 (0.0–30.0) [a]	30.0 (13.1–39.0) [b]	<0.001
Milk and dairies (g/day)	128 (67–149) [a]	1459 (99–188) [ab]	167 (145–211) [b]	0.002	129 (77–148) [a]	129 (79–168) [ab]	146 (121–189) [b]	0.009
Meat and poultry (g/day)	66 (32–95)	54 (27–79)	45 (27–65)	0.057	77 (41–100)	60 (34–88)	61 (42–88)	0.295
Wine (g/day)	0.0 (0.0–0.0)	0.0 (0.0–0.0)	0.0 (0.0–0.0)	1.000	0.0 (0.0–0.0)	0.0 (0.0–0.0)	0.0 (0.0–0.0)	1.000
Monounsaturated fat: saturated fat ratio	1.2 (1.1–1.3) [a]	1.3 (1.1–1.4) [b]	1.4 (1.3–1.5) [b]	<0.001	1.2 (1.0–1.3) [a]	1.3 (1.1–1.5) [b]	1.4 (1.3–1.5) [b]	<0.001

[1] Differences among adherence to the MD groups within each season (Kruskal-Wallis non-parametric one-way ANOVA for independent samples with multiple pairwise comparisons, $p < 0.05$). Different letters in the same raw indicate significant differences among MD groups (a < b < c). Values are median (25th–75th percentile).

Table S2. Carbon footprint (CF) and ecological footprint (EF) by seasons for each food groups.

Food Group	CF Winter g CO_2eq/day	CF Spring g CO_2eq/day	p [1]	EF Winter m^2/day	EF Spring m^2/day	p [1]
Meat & cured meat	517 (114–910)	547 (194–983)	0.057	2.42(0.3–4.9)	2.9 (0.8–5.4)	0.027
Poultry	0.0 (0.0–121.9)	93.8 (0.0–121.9)	0.008	0.0 (0.0–1.3)	1.0 (0.0–1.3)	0.008
Fish & shellfish	109.2 (0.0–193.2)	0.0 (0.0–109.2)	<0.001	2.4 (0.0–3.6)	0.0 (0.0–2.4)	<0.001
Eggs	12.4 (4.5–60.9)	8.7 (0.0–68.2)	0.791	0.1 (0.0–0.3)	0.0 (0.0–0.3)	0.796
Milk & dairies	327 (213–489)	296 (205–460)	0.534	1.9 (1.4–2.8)	1.8 (1.2–2.7)	0.764
Animal fats	0.0 (0.0–8.3)	0.0 (0.0–8.3)	0.093	0.0 (0.0–0.1)	0.0 (0.0–0.1)	0.064
Vegetable oils	40 (30–51)	40 (30–49)	0.428	0.6 (0.4–0.8)	0.6 (0.4–0.7)	0.430
Legumes	0.0 (0.0–0.0)	0.0 (0.0–16.6)	0.001	0.0 (0.0–0.0)	0.0 (0.0–0.2)	0.001
Potatoes	32.1 (0.0–52.2)	12.1 (0.0–67.1)	0.954	0.1 (0.0–0.2)	0.1 (0.0–0.3)	0.936
Cereals	173 (137–222)	175 (130–230)	0.612	1.0 (0.7–1.2)	0.9 (0.6–1.1)	0.031
Breakfast cereals	0.0 (0.0–34.2)	0.0 (0.0–34.2)	0.732	0.0 (0.0–0.1)	0.0 (0.0–0.1)	0.729
Bread & substitutes	44 (23–68)	32 (16–56)	0.010	0.3 (0.1–0.4)	0.2 (0.1–0.3)	0.005
Vegetables	57 (30–90)	51 (28–76)	0.005	0.2 (0.1–0.3)	0.2 (0.1–0.3)	0.007
Fruit	55 (31–95)	41 (18–70)	<0.001	0.3 (0.2–0.6)	0.3 (0.1–0.4)	<0.001
Nuts	0.0 (0.0–0.0)	0.0 (0.0–0.0)	0.180	0.0 (0.0–0.0)	0.0 (0.0–0.0)	0.180
Juices	44.7 (0.0–89.3)	0.0 (0.0–44.7)	0.008	0.7 (0.0–1.5)	0.0 (0.0–0.7)	0.014
Sweets & desserts	114 (72–201)	103 (58–160)	0.012	0.7 (0.5–1.3)	0.7 (0.4–1.0)	0.010
Pizza	326.1 (0.0–652.2)	217.4 (0.0–652.2)	0.008	1.2 (0.0–2.5)	0.8 (0.0–2.5)	0.008
Others	20.5 (0.0–88.9)	16.6 (0.0–75.2)	0.135	0.1 (0.0–0.6)	0.1 (0.0–0.4)	0.217
Tea & coffee decaffeinated	0.0 (0.0–0.0)	0.0 (0.0–0.0)	0.002	0.0 (0.0–0.0)	0.0 (0.0–0.0)	0.002
Soft drinks	0.0 (0.0–0.0)	0.0 (0.0–36.1)	0.102	0.0 (0.0–0.0)	0.0 (0.0–0.0)	1.000

[1] Between-season differences (non-parametric Wilcoxon Signed Ranks Test for paired samples, $p < 0.05$). Values are median (25th–75th percentile).

Table S3. Energy-adjusted carbon footprint (CF) and ecological footprint (EF) by seasons for each food groups.

Food Group	CF Winter g CO_2eq/1000 kcal	CF Spring g CO_2eq/1000 kcal	p [1]	EF Winter m^2/1000 kcal	EF Spring m^2/1000 kcal	p [1]
Meat & cured meat	315 (80–573)	377 (141–683)	0.005	1.5 (0.2–3.1)	2.0 (0.5–3.9)	0.003
Poultry	0.0 (0.0–75.9)	56.8 (0.0–98.9)	0.001	0.0 (0.0–0.8)	0.6 (0.0–1.1)	0.001
Fish & shellfish	67.3 (0.0–119.9)	0.0 (0.0–73.1)	<0.001	1.5 (0.0–2.5)	0.0 (0.0–1.6)	<0.001
Eggs	8.2 (2.9–35.9)	6.8 (0.0–42.7)	0.774	0.0 (0.0–0.2)	0.0 (0.0–0.2)	0.774
Milk & dairies	215 (141–304)	213 (157–323)	0.117	1.2 (0.8–1.8)	1.3 (0.9–1.9)	0.058
Animal fats	0.0 (0.0–5.7)	0.0 (0.0–5.4)	0.370	0.0 (0.0–0.1)	0.0 (0.0–0.1)	0.263
Vegetable oils	27 (19–34)	28 (22–34)	0.208	0.4 (0.3–0.5)	0.4 (0.3–0.5)	0.208
Legumes	0.0 (0.0–0.0)	0.0 (0.0–10.8)	0.001	0.0 (0.0–0.0)	0.0 (0.0–0.1)	<0.001
Potatoes	20.6 (0.0–31.4)	9.9 (0.0–47.4)	0.378	0.1 (0.0–0.1)	0.0 (0.0–0.2)	0.369
Cereals	113 (89–144)	126 (91–153)	0.009	0.6 (0.5–0.8)	0.6 (0.5–0.8)	0.811
Breakfast cereals	0.0 (0.0–26.1)	0.0 (0.0–24.3)	0.163	0.0 (0.0–0.1)	0.0 (0.0–0.1)	0.163
Bread & substitutes	27 (15–40)	23 (11–39)	0.076	0.2 (0.1–0.3)	0.1 (0.1–0.2)	0.074
Vegetables	37 (20–56)	35 (20–53)	0.110	0.1 (0.1–0.2)	0.1 (0.1–0.2)	0.136
Fruit	37 (20–62)	29 (14–50)	0.002	0.2 (0.1–0.4)	0.2 (0.1–0.3)	0.002
Nuts	0.0 (0.0–0.0)	0.0 (0.0–0.0)	0.040	0.0 (0.0–0.0)	0.0 (0.0–0.0)	0.040
Juices	28.5 (0.0–50.4)	0.0 (0.0–42.0)	0.090	0.5 (0.0–0.8)	0.0 (0.0–0.7)	0.090
Sweets & desserts	74 (48–123)	70 (46–111)	0.113	0.5 (0.3–0.8)	0.5 (0.3–0.7)	0.136
Pizza	191.7 (0.0–407.6)	144.8 (0.0–403.1)	0.065	0.7 (0.0–1.5)	0.5 (0.0–1.5)	0.065
Others	12.8 (0.0–51.4)	14.2 (0.0–51.3)	0.420	0.0 (0.0–0.4)	0.0 (0.0–0.4)	0.515
Tea & coffee decaffeinated	0.0 (0.0–0.0)	0.0 (0.0–0.0)	0.003	0.0 (0.0–0.0)	0.0 (0.0–0.0)	0.492
Soft drinks	0.0 (0.0–19.5)	0.0 (0.0–9.9)	0.206	0.0 (0.0–0.0)	0.0 (0.0–0.0)	1.000

[1] Between-season differences (non-parametric Wilcoxon Signed Ranks Test for paired samples, $p < 0.05$). Values are median (25th–75th percentile).

Table S4. Total and food-source carbon footprint (CF) and ecological footprint (EF) for each food groups among adherence to the Mediterranean Diet groups by seasons.

Source	Low MDS (n = 46)	Medium MDS (n = 80)	High MDS (n = 46)	p [1]	Low MDS (n = 39)	Medium MDS (n = 76)	High MDS (n = 55)	p [1]
	**CF Winter g CO_2eq/day**				**CF Spring g CO_2eq/day**			
Meat & cured meat	573 (160–931)	474 (114–900)	540 (77–877)	0.784	535 (252–1131)	604 (161–990)	545 (177–905)	0.690
Poultry	69.7 (0.0–121.9)	0.0 (0.0–121.9)	0.0 (0.0–97.2)	0.327	93.8 (0.0–215.7)	93.8 (0.0–121.9)	93.8 (0.0–121.9)	0.701
Fish & shellfish	22.8 (0.0–109.2) [a]	109.2 (0.0–141.9) [a]	188.0 (109.2–254.2) [b]	<0.001	0.0 (0.0–0.0) [a]	0 .0 (0.0–109.2) [a]	109.2 (65.6–142.6) [b]	<0.001
Eggs	6.2 (0.0–28.8) [a]	12.4 (6.2–68.2) [ab]	18.6 (6.2–74.4) [b]	0.004	7.4 (0.0–21.7) [a]	8.1 (0.0–28.8) [a]	21.1 (6.2–76.9) [b]	0.003
Milk & dairies	265 (187–339) [a]	341 (212–449) [a]	468 (312–618) [b]	<0.001	234 (190–329) [a]	340 (197–487) [b]	330 (250–474) [a]	0.026
Animal fats	1.7 (0.0–13.8)	0.0 (0.0–8.3)	0.0 (0.0–6.1)	0.192	0.0 (0.0–7.2)	0.0 (0.0–8.3)	0.0 (0.0–6.2)	0.587
Vegetable oils	31 (20–40) [a]	41 (30–51) [b]	49 (38–63) [c]	<0.001	30 (21–43) [a]	41 (28–51) [b]	42 (38–51) [b]	<0.001
Legumes	0.0 (0.0–0.0) [a]	0.0 (0.0–0.0) [ab]	0.0 (0.0–18.0) [b]	0.021	0.0 (0.0–0.0) [a]	0.0 (0.0–16.6) [ab]	5.5 (0.0–27.7) [b]	0.002
Potatoes	1.9 (0.0–40.2) [a]	37.2 (2.0–63.3) [b]	32.1 (1.5–55.7) [ab]	0.008	8.0 (0.0–66.1)	10.5 (0.0–67.1)	28.1 (4.9–67.1)	0.139
Cereals	160 (109–205)	174 (144–219)	187 (144–229)	0.147	155 (106–221)	160 (124–224)	193 (145–236)	0.070
Breakfast cereals	0.0 (0.0–45.6)	0.0 (0.0–34.2)	0.0 (0.0–37.1)	0.612	0.0 (0.0–34.2)	0.0 (0.0–34.2)	0.0 (0.0–34.2)	0.739
Bread & substitutes	45 (23–79)	35 (18–64)	47 (31–68)	0.186	22 (14–35) [a]	37 (16–59) [b]	35 (17–71) [b]	0.008
Vegetables	30 (13–52) [a]	55 (31–87) [b]	94 (69–109) [c]	<0.001	35 (17–49) [a]	48 (26–76) [b]	70 (52–84) [c]	<0.001
Fruit	32 (12–51) [a]	53 (33–93) [b]	90 (72–114) [c]	<0.001	24.3 (0.8–53.3) [a]	32.2 (10.1–57.2) [a]	63.4 (41.1–90.0) [b]	<0.001
Nuts	0.0 (0.0–0.0)	0.0 (0.0–0.0)	0.0 (0.0–0.0)	0.064	0.0 (0.0–0.0)	0.0 (0.0–0.0)	0.0 (0.0–0.0)	0.064
Juices	44.7 (0.0–89.3)	44.7 (0.0–89.3)	44.7 (0.0–81.0)	0.754	0.0 (0.0–58.1)	0.0 (0.0–44.7)	0.0 (0.0–78.2)	0.680
Sweets & desserts	97 (65–178)	113 (72–213)	137 (82–201)	0.433	112 (69–164)	78 (55–155)	112 (72–186)	0.163
Pizza	326.1 (0.0–652.2)	326.1 (0.0–652.2)	326.1 (0.0–652.2)	0.963	217.4 (0.0–326.1)	163.0 (0.0–652.2)	326.1 (0.0–6522)	0.367
Others	16.6 (0.0–124.3)	24.1 (0.0–79.1)	18.1 (0.0–80.9)	0.775	16.6 (0.0–51.3)	16.6 (0.0–107.4)	20.5 (0.0–52.4)	0.909
Tea & coffee decaffeinated	0.0 (0.0–0.0)	0.0 (0.0–0.0)	0.0 (0.0–0.0)	0.676	0.0 (0.0–0.0) [ab]	0.0 (0.0–0.0) [a]	0.0 (0.0–0.0) [b]	0.016
Soft drinks	0.0 (0.0–36.1)	0.0 (0.0–36.1)	0.0 (0.0–36.1)	0.545	0.0 (0.0–27.3)	0.0 (0.0–0.0)	0.0 (0.0–27.6)	0.563
	**EF Winter m^2/day**				**EF Spring m^2/day**			
Meat & cured meat	2.9 (0.4–5.0)	2.3 (0.3–4.9)	2.7 (0.2–4.6)	0.809	2.9 (1.2–6.9)	3.1 (0.5–5.3)	2.8 (0.7–4.8)	0.616
Poultry	0.8 (0.0–1.3)	0.0 (0.0–1.3)	0.0 (0.0–1.1)	0.327	1.0 (0.0–2.3)	1.0 (0.0–1.3)	1.0 (0.0–1.3)	0.701
Fish & shellfish	0.5 (0.0–2.4) [a]	2.4 (0.0–3.1) [a]	3.9 (2.4–5.5) [b]	<0.001	0.0 (0.0–0.0) [a]	0.0 (0.0–2.4) [a]	2.4 (1.0–3.1) [b]	<0.001
Eggs	0.0 (0.0–0.1) [a]	0.1 (0.0–0.3) [ab]	0.1 (0.0–0.3) [b]	0.004	0.0 (0.0–0.1) [a]	0.0 (0.0–0.1) [a]	0.1 (0.0–0.3) [b]	0.003
Milk & dairies	1.5 (1.1–2.0) [a]	1.9 (1.2–2.7) [a]	2.5 (1.6–3.6) [b]	<0.001	1.4 (1.1–1.9) [a]	1.9 (1.1–3.0) [ab]	1.9 (1.5–2.6) [b]	0.038
Animal fats	0.0 (0.0–0.1)	0.0 (0.0–0.1)	0.0 (0.0–0.1)	0.225	0.0 (0.0–0.1)	0.0 (0.0–0.1)	0.0 (0.0–0.1)	0.665
Vegetable oils	0.4 (0.3–0.6) [a]	0.6 (0.4–0.8) [b]	0.7 (0.5–0.9) [c]	<0.001	0.4 (0.3–0.6) [a]	0.6 (0.4–0.8) [b]	0.6 (0.5–0.8) [b]	<0.001

Legumes	0.0 (0.0–0.0) [a]	0.0 (0.0–0.0) [ab]	0.0 (0.0–0.2) [b]	0.021	0.0 (0.0–0.0) [a]	0.0 (0.0–0.2) [ab]	0.1 (0.0–0.3) [b]	0.002
Potatoes	0.0 (0.0–0.2) [a]	0.2 (0.0–0.2) [b]	0.1 (0.0–0.2) [ab]	0.008	0.0 (0.0–0.3)	0.0 (0.0–0.3)	0.1 (0.0–0.3)	0.150
Cereals	0.9 (0.6–1.1)	0.9 (0.7–1.2)	1.0 (0.8–1.2)	0.174	0.8 (0.6–1.2) [a]	0.8 (0.6–1.1) [ab]	0.9 (0.7–1.2) [b]	0.049
Breakfast cereals	0.0 (0.0–0.2)	0.0 (0.0–0.1)	0.0 (0.0–0.1)	0.612	0.0 (0.0–0.1)	0.0 (0.0–0.1)	0.0 (0.0–0.1)	0.739
Bread & substitutes	0.3 (0.2–0.4)	0.2 (0.1–0.4)	0.3 (0.2–0.4)	0.154	0.1 (0.1–0.2) [a]	0.2 (0.1–0.4) [b]	0.2 (0.1–0.4) [b]	0.010
Vegetables	0.1 (0.0–0.2) [a]	0.2 (0.1–0.3) [b]	0.3 (0.2–0.4) [c]	<0.001	0.1 (0.1–0.2) [a]	0.2 (0.1–0.3) [b]	0.2 (0.2–0.3) [b]	<0.001
Fruit	0.2 (0.1–0.3) [a]	0.3 (0.2–0.6) [b]	0.6 (0.4–0.7) [c]	<0.001	0.1 (0.0–0.3) [a]	0.2 (0.1–0.4) [a]	0.4 (0.3–0.6) [b]	<0.001
Nuts	0.0 (0.0–0.0)	0.0 (0.0–0.0)	0.0 (0.0–0.0)	0.064	0.0 (0.0–0.0)	0.0 (0.0–0.0)	0.0 (0.0–0.0)	0.064
Juices	0.7 (0.0–1.5)	0.7 (0.0–1.5)	0.7 (0.0–1.3)	0.754	0.0 (0.0–1)	0.0 (0.0–0.7)	0.0 (0.0–1.3)	0.680
Sweets & desserts	0.6 (0.4–1.1)	0.7 (0.5–1.3)	0.9 (0.5–1.3)	0.429	0.8 (0.4–1.1)	0.5 (0.4–1.0)	0.8 (0.5–1.3)	0.076
Pizza	1.2 (0.0–2.5)	1.2 (0.0–2.5)	1.2 (0.0–2.5)	0.963	0.8 (0.0–1.2)	0.6 (0.0–2.5)	1.2 (0.0–2.5)	0.367
Others	0.1 (0.0–1.0)	0.1 (0.0–0.2)	0.1 (0.0–0.4)	0.473	0.1 (0.0–0.3)	0.1 (0.0–0.9)	0.1 (0.0–0.2)	0.843
Tea & coffee decaffeinated	0.0 (0.0–0.0)	0.0 (0.0–0.0)	0.0 (0.0–0.0)	0.685	0.0 (0.0–0.0) [ab]	0.0 (0.0–0.0) [a]	0.0 (0.0–0.0) [b]	0.016
Soft drinks	0.0 (0.0–0.0)	0.0 (0.0–0.0)	0.0 (0.0–0.0)	1.000	0.0 (0.0–0.0)	0.0 (0.0–0.0)	0.0 (0.0–0.0)	1.000

[1] Differences among adherence to the MD groups within each season (Kruskal-Wallis non-parametric one-way ANOVA for independent samples with multiple pairwise comparisons, $p < 0.05$). Different letters in the same raw indicate significant differences among MD groups (a < b < c). Values are median (25th–75th percentile).

Table S5. Energy-adjusted (per 1000 kcal) total and food–source carbon footprint (CF) and ecological footprint (EF) for each food groups among adherence to the Mediterranean Diet groups by seasons.

**Source**	**Low MDS (*n* = 44)**	**Medium MDS (*n* = 80)**	**High MDS (*n* = 46)**	***p*** [1]	**Low MDS (*n* = 39)**	**Medium MDS (*n* = 76)**	**High MDS (*n* = 55)**	***p*** [1]
	**CF Winter g CO_2eq/1000 kcal**				**CF Spring g CO_2eq/1000 kcal**			
Meat & cured meat	394 (116–627)	315 (80–562)	296 (42–530)	0.367	467 (178–912)	402 (120–651)	320 (133–624)	0.155
Poultry	48.1 (0.0–88.8)	0.0 (0.0–76.8)	0.0 (0.0–60.7)	0.183	69.2 (0.0–149.9)	56.8 (0.0–96.1)	55.1 (0.0–95.3)	0.779
Fish & shellfish	15.3 (0.0–75.6) [a]	62.6 (0.0–117.8) [a]	103.5 (61.3–175.0) [b]	<0.001	0.0 (0.0–0.0) [a]	0.0 (0.0–65.4) [a]	68.4 (45.3–97.6) [b]	<0.001
Eggs	3.9 (0.0–21.6) [a]	8.3 (3.7–37.1) [ab]	10.4 (4.1–40.8) [b]	0.026	5.5 (0.0–20.0) [a]	6.3 (0.0–18.8) [a]	11.5 (4.2–48.8) [b]	0.033
Milk & dairies	188 (117–251) [a]	215 (144–293) [ab]	274 (168–372) [b]	0.008	197 (155–304)	223 (141–357)	214 (165–296)	0.699
Animal fats	1.3 (0.0–9.3)	0.0 (0.0–6.0)	0.0 (0.0–3.8)	0.122	0.0 (0.0–5.0)	0.0 (0.0–5.9)	0.0 (0.0–3.9)	0.450
Vegetable oils	22 (14–30) [a]	29 (19–34) [b]	32 (22–38) [b]	<0.001	25 (19–30)	29 (20–35)	29 (22–34)	0.106
Legumes	0.0 (0.0–0.0) [a]	0.0 (0.0–0.0) [ab]	0.0 (0.0–9.8) [b]	0.022	0.0 (0.0–0.0) [a]	0.0 (0.0–13.8) [ab]	3.4 (0.0–17) [b]	0.003
Potatoes	1.3 (0.0–27.2) [a]	25.3 (1.1–42.8) [b]	18.5 (0.8–31.2) [ab]	0.009	4.5 (0.0–45.3)	8.0 (0.0–48.8)	18.8 (3.1–49.6)	0.319
Cereals	108 (79–141)	112 (92–150)	113 (92–141)	0.560	138 (87–190)	125 (88–152)	124 (94–151)	0.751
Breakfast cereals	0.0 (0.0–26.8)	0.0 (0.0–25.9)	0.0 (0.0–23.5)	0.646	0.0 (0.0–34.2)	0.0 (0.0–22.8)	0.0 (0.0–23.8)	0.814
Bread & substitutes	31 (17–56)	25 (12–37)	29 (16–40)	0.328	18 (11–28)	23 (12–47)	28 (11–42)	0.123
Vegetables	19.9 (8.3–34.2) [a]	37.2 (20.7–58.2) [b]	54.0 (40.1–62.3) [c]	<0.001	25 (16–35) [a]	34 (18–54) [a]	44 (32–55) [b]	<0.001
Fruit	22.4 (7.6–38.1) [a]	35.4 (19.4–60.9) [b]	55.6 (39.6–69.5) [c]	<0.001	22.4 (0.5–42.2) [a]	20.4 (7.7–40.7) [a]	41.0 (28.0–57.6) [b]	<0.001
Nuts	0.0 (0.0–0.0)	0.0 (0.0–0.0)	0.0 (0.0–0.0)	0.070	0.0 (0.0–0.0)	0.0 (0.0–0.0)	0.0 (0.0–0.0)	0.078
Juices	29.4 (0.0–58.6)	28.8 (0.0–54.2)	27.5 (0.0–45.3)	0.949	0.0 (0.0–47.4)	0.0 (0.0–35)	0.0 (0.0–41.9)	0.863
Sweets & desserts	78 (40–115)	72 (49–132)	69 (51–114)	0.931	89 (57–115)	60 (34–110)	71 (50–102)	0.061
Pizza	214.2 (0.0–472.6)	194.4 (0.0–432.4)	182.5 (0.0–356.5)	0.778	143.0 (0.0–319.3)	127.5 (0.0–445.4)	161.3 (0.0–381.4)	0.752
Others	12.1 (0.0–82.2)	15.6 (0.0–45.3)	10.8 (0.0–42.4)	0.615	16.5 (0.0–36.9)	13.8 (0.0–71.3)	12.6 (0.0–33.9)	0.797
Tea & coffee decaffeinated	0.0 (0.0–0.0)	0.0 (0.0–0.0)	0.0 (0.0–0.0)	0.663	0.0 (0.0–0.0) [ab]	0.0 (0.0–0.0) [a]	0.0 (0.0–0.0) [b]	0.016
Soft drinks	0.0 (0.0–20.4)	0.0 (0.0–20.6	0.0 (0.0–16.9)	0.692	0.0 (0.0–24.7)	0.0 (0.0–0.0)	0.0 (0.0–16.4)	0.375
	**EF Winter m^2/1000 kcal**				**EF Spring m^2/1000 kcal**			
Meat & cured meat	1.9 (0.3–3.5)	1.6 (0.2–3.1)	1.4 (0.1–2.7)	0.391	2.8 (1.0–5.2)	2.1 (0.4–3.8)	1.7 (0.5–3.5)	0.156
Poultry	0.5 (0.0–1.0)	0.0 (0.0–0.8)	0.0 (0.0–0.7)	0.183	0.7 (0.0–1.6)	0.6 (0.0–1.0)	0.6 (0.0–1.0)	0.779
Fish & shellfish	0.3 (0.0–1.6) [a]	1.4 (0.0–2.4) [a]	2.2 (1.3–3.6) [b]	<0.001	0.0 (0.0–0.0) [a]	0.0 (0.0–1.4) [a]	1.5 (0.6–2.0) [b]	<0.001
Eggs	0.0 (0.0–0.1) [a]	0.0 (0.0–0.2) [ab]	0.0 (0.0–0.2) [b]	0.026	0.0 (0.0–0.1) [a]	0.0 (0.0–0.1) [a]	0.0 (0.0–0.2) [b]	0.033
Milk & dairies	1.1 (0.7–1.4) [a]	1.2 (0.9–1.8) [ab]	1.5 (0.9–2.2) [b]	0.015	1.2 (0.9–1.8)	1.3 (0.8–2.2)	1.3 (1.0–1.7)	0.744
Animal fats	0.0 (0.0–0.1)	0.0 (0.0–0.1)	0.0 (0.0–0.0)	0.148	0.0 (0.0–0.0)	0.0 (0.0–0.1)	0.0 (0.0–0.0)	0.489
Vegetable oils	0.3 (0.2–0.4) [a]	0.4 (0.3–0.5) [b]	0.5 (0.3–0.5) [b]	<0.001	0.4 (0.3–0.4)	0.4 (0.3–0.5)	0.4 (0.3–0.5)	0.106

Legumes	0.0 (0.0–0.0) [a]	0.0 (0.0–0.0) [ab]	0.0 (0.0–0.1) [b]	0.022	0.0 (0.0–0.0) [a]	0.0 (0.0–0.1) [ab]	0.0 (0.0–0.2) [b]	0.003
Potatoes	0.0 (0.0–0.1) [a]	0.1 (0.0–0.2) [b]	0.1 (0.0–0.1) [ab]	0.011	0.0 (0.0–0.2)	0.0 (0.0–0.2)	0.1 (0.0–0.2)	0.330
Cereals	0.6 (0.4–0.8)	0.6 (0.5–0.8)	0.6 (0.5–0.7)	0.641	0.6 (0.4–0.9)	0.6 (0.5–0.8)	0.6 (0.5–0.8)	0.879
Breakfast cereals	0.0 (0.0–0.1)	0.0 (0.0–0.1)	0.0 (0.0–0.1)	0.646	0.0 (0.0–0.1)	0.0 (0.0–0.1)	0.0 (0.0–0.1)	0.814
Bread & substitutes	0.2 (0.1–0.3)	0.2 (0.1–0.2)	0.2 (0.1–0.2)	0.291	0.1 (0.1–0.2)	0.1 (0.1–0.3)	0.2 (0.1–0.2)	0.125
Vegetables	0.1 (0.0–0.1) [a]	0.1 (0.1–0.2) [b]	0.2 (0.1–0.2) [c]	<0.001	0.1 (0.1–0.1) [a]	0.1 (0.1–0.2) [a]	0.2 (0.1–0.2) [b]	<0.001
Fruit	0.1 (0.0–0.2) [a]	0.2 (0.1–0.4) [b]	0.3 (0.2–0.4) [c]	<0.001	0.1 (0.0–0.3) [a]	0.1 (0.0–0.2) [a]	0.3 (0.2–0.4) [b]	<0.001
Nuts	0.0 (0.0–0.0)	0.0 (0.0–0.0)	0.0 (0.0–0.0)	0.070	0.0 (0.0–0.0)	0.0 (0.0–0.0)	0.0 (0.0–0.0)	0.078
Juices	0.5 (0.0–1.0)	0.5 (0.0–0.9)	0.5 (0.0–0.7)	0.949	0.0 (0.0–0.8)	0.0 (0.0–0.6)	0.0 (0.0–0.7)	0.863
Sweets & desserts	0.5 (0.3–0.7)	0.5 (0.3–0.8)	0.5 (0.3–0.7)	0.952	0.6 (0.4–0.8) [b]	0.4 (0.2–0.7) [a]	0.5 (0.3–0.7) [ab]	0.043
Pizza	0.8 (0.0–1.8)	0.7 (0.0–1.6)	0.7 (0.0–1.3)	0.778	0.5 (0.0–1.2)	0.5 (0.0–1.7)	0.6 (0.0–1.4)	0.752
Others	0.1 (0.0–0.7)	0.0 (0.0–0.2)	0.1 (0.0–0.2)	0.404	0.1 (0.0–0.2)	0 (0.0–0.5)	0.0 (0.0–0.1)	0.556
Tea & coffee decaffeinated	0.0 (0.0–0.0)	0.0 (0.0–0.0)	0.0 (0.0–0.0)	0.668	0.0 (0.0–0.0) [ab]	0.0 (0.0–0.0) [a]	0.0 (0.0–0.0) [b]	0.016
Soft drinks	0.0 (0.0–0.0)	0.0 (0.0–0.0)	0.0 (0.0–0.0)	1.000	0.0 (0.0–0.0)	0.0 (0.0–0.0)	0.0 (0.0–0.0)	1.000

[1] Differences among adherence to the MD groups within each season (Kruskal-Wallis non-parametric one-way ANOVA for independent samples with multiple pairwise comparisons, $p < 0.05$). Different letters in the same raw indicate significant differences among MD groups (a < b < c). Values are median (25th–75th percentile).
